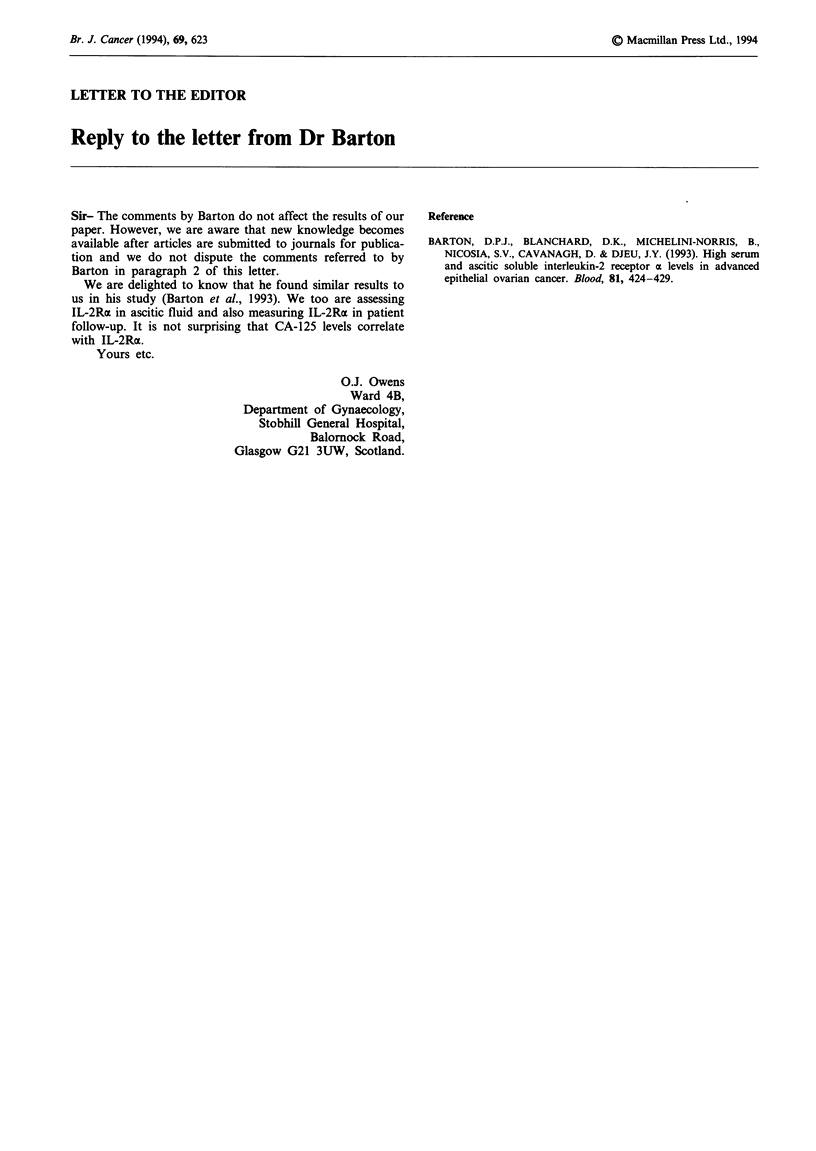# Reply to the letter from Dr Barton

**Published:** 1994-03

**Authors:** O.J. Owens


 © Macmillan Press Ltd., 1994

LETTER TO THE EDITOR

### Reply to the letter from Dr Barton

Sir– The comments by Barton do not affect the results of our paper. However, we are aware that new knowledge becomes available after articles are submitted to journals for publication and we do not dispute the comments referred to by Barton in paragraph 2 of this letter.

We are delighted to know that he found similar results to us in his study (Barton *et al.*, 1993). We too are assessing IL-2Rα in ascitic fluid and also measuring IL-2Rα in patient follow-up. It is not surprising that CA-125 levels correlate with IL-2Rα.

Yours etc.

O.J. Owens
Ward 4B,
Department of Gynaecology,
Stobhill General Hospital,
Balornock Road,
Glasgow G21 3UW, Scotland.

**Reference**

BARTON, D.P.J., BLANCHARD, D.K., MICHELINI-NORRIS, B., NICOSIA, S.V., CAVANAGH, D. & DJEU, J.Y. (1993). High serum and ascitic soluble interleukin-2 receptor α levels in advanced epithelial ovarian cancer. *Blood*, **81**, 424–429.